# Nanoscale Plasticity Behavior of Additive-Manufactured Zirconia-Toughened Alumina Ceramics during Nanoindentation

**DOI:** 10.3390/ma13041006

**Published:** 2020-02-24

**Authors:** Wenli Li, Weiwei Liu, Maoshan Li, Jianbin Nie, Yao Chen, Zhanwen Xing

**Affiliations:** School of Mechanical & Electric Engineering, Soochow University, Suzhou 215021, China; wlli@suda.edu.cn (W.L.); liuweiwei@suda.edu.cn (W.L.); 20185229059@stu.suda.edu.cn (M.L.); 20185229029@stu.suda.edu.cn (J.N.); chenyao@suda.edu.cn (Y.C.)

**Keywords:** additive manufacturing, stereolithography, zirconia-toughened alumina, nanoindentation, microcracking

## Abstract

The nanoscale plasticity phenomena in zirconia-toughened alumina (ZTA) ceramics with yttria-stabilized zirconia (YSZ) addition of 10% and 30% fabricated by additive manufacturing based on a stereolithography technique were explored in detail by nanoindentation and scanning electron microscopy. It was demonstrated that the initiation of nanoscale plasticity was attributed to the combined contributions from the generation of nanoscale shear deformation bands and localized microcracking at the indentations. Such localized plastic behavior underneath the nanoindenter was interpreted by maximum shear stress analysis. The response of the phase boundary during indentation was emphasized through crack propagation paths, and optimization of alumina–YSZ adaptation through component design and SL processing was expected.

## 1. Introduction

Due to the high hardness and biological inertia of alumina along with excellent toughness originating from the phase transformation of zirconia, increasing attention is being paid to zirconia-toughened alumina ceramics (ZTA), which are widely used in biomedical components and orthopedic implants [1,2,3]. Meanwhile, along with the development of additive-manufacturing (AM) techniques for ceramic materials, several technologies have been exploited to produce such customized biomedical ceramic parts instead of traditional precision machining [4,5,6,7]. In particular, stereolithography (SL) has received great interest due to its advantages of optimized surface quality and high precision [8]. For example, Chen et al. used microstereolithography to produce ZTA ceramics with slightly lower hardness and toughness than those produced by conventional methods [9], and dense ZTA ceramics were prepared by Wu et al. by SL, with properties comparable to traditional molding methods [10]. In short, these studies on SL of ZTA ceramics were based on photocuring of layer-by-layer recoated ceramic slurry with high fluidity, in which the support structure is essential to the construction of complex biomedical components with overhanging structures. Such support structures are detrimental to the surface quality during their subsequent removal. Recently, SL of ceramics at the paste stage was realized by the authors of the present research, which enhanced the feasibility of SL for ZTA biomedical components.

Meanwhile, all of the above-mentioned biomedical applications of SL-fabricated ZTA ceramics invariable involve contact-induced deformation and fracture [11], due to the challenge of intrinsic plasticity. In fact, while most ceramics have inherently brittle behavior, the plasticity behavior of structural ceramics has already become the subject of considerable interest [12], and dislocation configurations have been observed after indentation, scratch, and high-rate deformation processes [13,14,15]. However, for additive-manufactured ceramics, mechanical evaluation has been limited to static hardness, flexural strength, and fracture toughness [10,16,17,18]. The defects of additive-manufactured ZTA ceramics, which can initiate complete failure of the microstructural integrity, usually originate with localized deformation or damage during biomedical service processes [19]. Understanding the onset and mechanism of contact-induced deformation at the local scale is therefore necessary before the widespread application of SL-fabricated ZTA biomedical parts. In fact, local stress is imposed on very small areas of the ceramic surface in real contact situations. Due to the inherent brittleness of ceramics, localized deformation or fracture can initiate premature failure during the biomedical component’s service life. Understanding the onset of local plasticity behavior for these additive-manufactured ZTA ceramics is therefore necessary, and both macro- and micro-mechanical behaviors should be synthetically considered for optimization of component design for SL and related processing factors. In fact, several studies have reported the extent of plasticity in ceramics by the method of nanoindentation [3,12,20,21], and indentation-induced damage has been tracked under different normal loads [22]. Additionally, it is expected that the deformation behavior of zirconia and alumina will be different in a zirconia–alumina composite. Whether or not such discrepancies can be displayed at a localized scale under contact-induced deformation is in doubt. Certainly, instead of instrumented indentation, the localized deformation behavior of each phase in a composite system cannot be distinguished directly through macro-mechanical testing.

The present work mainly reports experimental observations of nanoscale plasticity behavior in ZTA ceramics fabricated by SL-based AM technique from the form of ceramic paste. Maximum shear stress analysis was interpreted with nanoindentation under a variety of applied loads. Additionally, the associated objective was to identify differences between the phases with corresponding plasticity mechanism initiates during indentation. The results facilitate comprehension of the localized deformation behaviors of additive-manufactured ZTA ceramics and provide suggestions for further application of these as-processed components.

## 2. Materials and Methods

Ceramic pastes were prepared by mixing photoinitiator, dispersant, plasticizer, acrylate monomers, and ceramic powder, wherein the mass ratios of yttria-stabilized zirconia (YSZ) to alumina were 10:90 (ZTA10) and 30:70 (ZTA30), and all components were mixed by a magnetic stirrer. A commercialized manufacturing system (AMC150, ZRapid Tech., Suzhou, China) was used to obtain SL green samples with a preset layer thickness of 60 m. The as-printed green bodies were debound at 600 °C and finally sintered at 1580 °C. The nanohardness (H) and Young’s modulus (E) of the sintered ZTA samples were evaluated by load-controlled nanoindentation equipment (NHT2, CSM, Peseux, Switzerland). A matrix form (5 × 5) of indentations was organized for each applied load, with an interval of 40 m between adjacent imprints. The tip radius of the Berkovich indenter was ~150 nm. H and E values of tested ZTA samples were analyzed by the Oliver–Pharr method [23]. During nanoindentation, a series of peak loads (10, 30, 50, 70, 100, 200, 300, 400, and 500 mN) were applied. To ascertain morphologies of the green bodies and sintered parts of ZTA10 and ZTA30 along with the imprints, scanning electron microscopy (SEM, SU-5000, HITACHI, Tokyo, Japan) technique was used with both secondary electron and backscattered electron modes.

## 3. Results and Discussions

**Figure 1:**
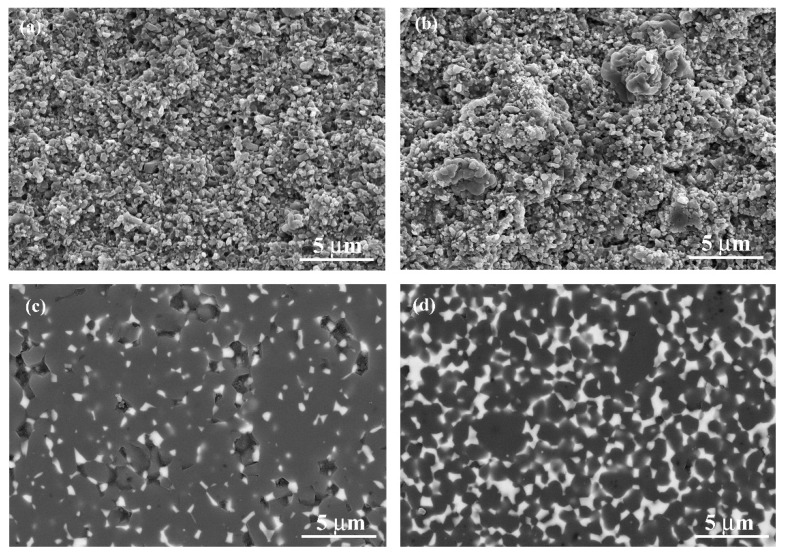
(**a**,**b**) Scanning electron microscopy (SEM) images of printed ZTA10 and ZTA30 green bodies, respectively; (**c**,**d**) backscattered electron images of sintered ZTA10 and ZTA30 ceramics, respectively.

The morphologies of as-printed green bodies of ZTA10 and ZTA30 are shown in Figure 1a,b. Most of the powder particles were entirely covered with cured resin, while abundant amount of cured resin and retained plasticizer were expected. It was evident that agglomeration tended to develop with higher contents of YSZ (Figure 1b). After sintering (Figure 1c,d), the microstructures of ZTA samples appeared much denser, with YSZ grains locating at grain boundaries or triple junctions of alumina grains. Additionally, in comparison to the sintered ZTA10 and ZTA30 shown in Figure 1c,d, the refinement of alumina grain size should not be ignored, considering the beneficial effect of YSZ dispersion in the matrix [24]. The homogenization of the size distribution and the refinement of the average size of the matrix was confirmed by quantitative analysis, where average grain sizes of alumina were 1.45 ± 0.04 m and 1.09 ± 0.01 m for ZTA10 and ZTA30, respectively. With increasing content of YSZ, the average grain size varied from 0.29 ± 0.07 m to 0.41 ± 0.08 m, implying the moderate coarsening of the YSZ grains. Such refinement of the alumina matrix can improve macroscale properties, especially flexural strength and fracture toughness. The microscale level displayed different features, as verified by subsequent tests.

**Figure 2:**
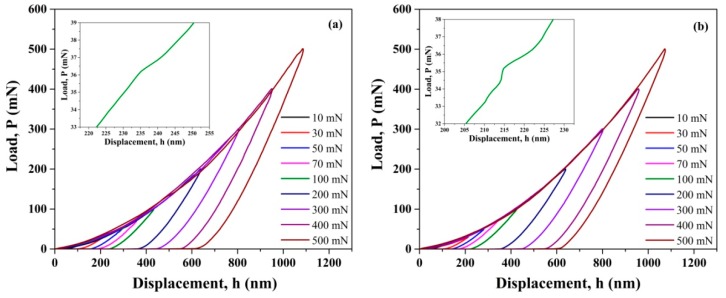
Typical P-h curves with different peak loads: (**a**) ZTA10; (**b**) ZTA30. The left inset of each figure presents the magnified serration.

The typical load (P) versus displacement (h) profiles for a variety of peak loads are demonstrated in Figure 2. All P-h plots implied elasto-plastic behaviors, in accordance with the conventional characteristics of ceramics [2,3]. Statically, indentation size effect could be observed at very low loads, while at higher loads above 50 mN, average values of both H and E stabilized. For the as-sintered ZTA10 ceramics, the average H value was ~22.23 ± 1.36 GPa and E was ~345.55 ± 17.63 GPa; correspondingly, average H and E values for ZTA30 were ~22.94 ± 0.72 GPa and 344.92 ± 16.23 GPa, respectively. It is likely that lower hardness values could be obtained for ZTAs with higher content of YSZ grains. In fact, the difference was not remarkable in H and E values between ZTA10 and ZTA30, although the contents of the YSZ phase were significantly different. Although stress-induced phase transformation leads to volume expansion in ZTA ceramics [25], it did not cause degradation of nanohardness at the present work. It was inferred that the densified sizes of sintered ZTA10 and ZTA30 compared to green bodies, the tip radius of the nanoindenter, applied load ranges, and therefore the localized areas under the indentation tip were possible contributing factors. Insets in Figure 2 are enlarged to show the particular serrations of the loading parts of the P-h plots at a lower peak load of 100 mN. Such serrations during the loading process suggest the initiation of localized plasticity events. According to each P-h curve, the critical load (P_c_) is the load at and above which nanoscale plasticity can be stimulated in ceramics due to the initiation of dislocations [26]. Statistical analysis revealed that Pc of ZTA10 changed from 0.06 to 1.55 mN under different peak loads, which was comparable to the results of ZTA30 (in the range of 0.04–1.82 mN).

**Figure 3:**
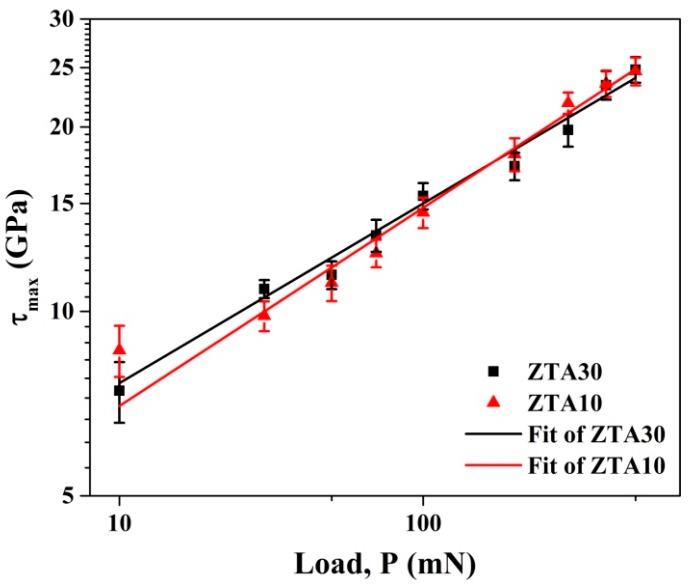
Maximum shear stress (τ_max_) variation underneath the nanoindentation tip with applied peak load.

Previous studies have implied that the load required to initiate dislocation-related plasticity can be calculated on the basis of the fitting by Hertzian elastic contact solution during nanoindentation experiments [26]. For each peak load, the maximum shear stress (τ_max_) operative under the nanoindentation tip is calculated according to method presented in Reference [19]. The estimated magnitude of τ_max_ for ZTA10 increased from about 8.64 to 24.66 GPa, which was far above the typical theoretical shear stress (τ_theo_) of 2.48 GPa of ZTA10 ceramics [27]. Meanwhile, the value for ZTA30 increased from 7.42 to 24.81 GPa, while τ_theo_ was as low as 1.93 GPa. Therefore, shear-induced deformation band and/or microcracking can be initiated by the sufficient stress during nanoindentation of ZTA10 and ZTA30 when τ_max_ is ≥τ_theo_ [28], and the initiation of localized plasticity events was observed throughout the loading process. Similarly to the empirical power law dependence of τ_max_ with P_c_ [29], a linear dependence of τ_max_ on P_c_ (Figure 3) was expected in the present work. Further evidence from SEM studies supported this speculation. Table 1 lists the comparison of related variations for ZTA ceramics manufactured by AM and conventional technology. It was concluded that within the load range of 10 mN to 500 mN, according to this work and Reference [30], τ_max_ varied at a similar level, while h_c_ (critical depth of penetration) and P_c_ deviated from each other after fitting.

**Figure 4:**
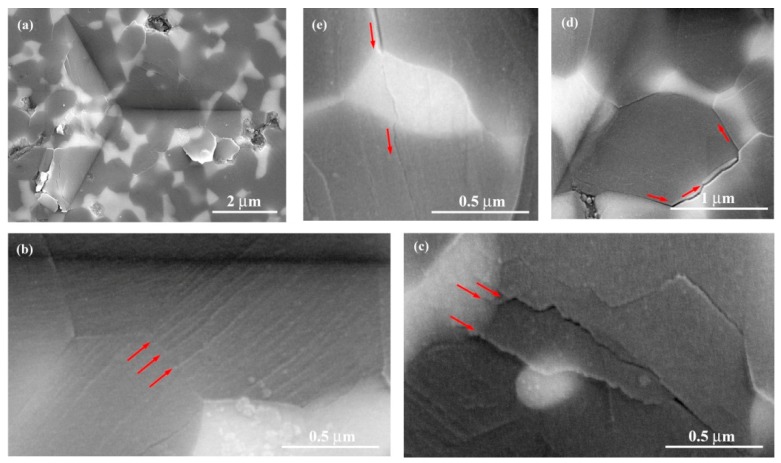
SEM images of shear deformation bands and microcracking formation inside the nanoindentation imprint under peak load of 500 mN for ZTA30: (**a**) typical overview of the imprint; (**b**) magnified view of shear deformation band group denoted by parallel arrows; (**c**) propagation of microcracking along shear deformation band; (**d**) intergranular fracture along alumina/YSZ boundaries; (**e**) transgranular fracture across the YSZ grain.

Since the sintered ZTA10 and ZTA30 samples fabricated by SL technique acted similarly in the aforementioned P-h analysis and shear stress estimation, typical morphologies of imprint at ZTA30 were chosen to elaborate the deformation mechanism under indentation (Figure 4). Various shear deformation bands developed at the alumina grain inside the imprint, as marked by parallel arrows in Figure 4b. Indeed, the intensity of shear deformation bands (i.e., the extent of plastic deformation) differed from individual grains, in which the intervals between two successive shear deformation bands were between 25 and 100 nm. It was verified that the shear deformation band interval spacing was closely associated to the localized microstructure of the composite, the magnitude of applied load, difficulty of shear deformation, etc. [30]. Additionally, a localized zone of weakness remained with pre-existing flaws during processing, which may have behaved to prioritize shear deformation [31], despite the fact that no pores or cracks appeared in the particular imprint of ZTA30 in Figure 4a. Meanwhile, microcracking prevailed at both alumina and YSZ grains inside the indents (Figure 4c–e). Theoretically, a low value of the critical resolved shear stress (τ_CRSS_) is required for microcracking formation. By means of estimation of τ_CRSS_ according to the approach suggested in Reference [32], along with the as-received experimental results, small magnitudes of average τ_CRSS_ (<1 GPa) for both ZTA10 and ZTA30 were determined. Therefore, owing to high stresses concentrated at a small contact region, a high probability of localized microcracking at the imprints was indicated.

It was obvious that the crack propagation path reflected a few regional deflections throughout the YSZ grain in Figure 4c–e. Similarly to crack deflection behavior during macro-mechanical testing of ZTA ceramics [33], such a mechanism also operated at the localized area of imprint in the present case, while intergranular and transgranular propagation paths existed simultaneously. The reason for this phenomenon involves several factors, particularly the appropriate adaptation of YSZ and alumina grains, including uniform distribution with comparable sizes at the composite after SL fabrication of the as-prepared ceramic paste. Concerning the phase boundary effect during the localized deformation, it was confirmed that yielding behavior tended to be stimulated easily at grain/phase boundaries of composites compared to grain interiors [25]. While the microcracks prevailed, as shown in Figure 4c,e, the situation in Figure 4d appeared particularly with cracks propagating along the alumina/YSZ boundaries, which was attributed to the phase boundaries with more open structure than that of a perfect lattice [25]. Although it appeared that simultaneous occurrences of both nanoscale plasticity and microcracking played a part under nanoindentation, remaining problems for future research include the detailed improvement of component design and SL processing to fully demonstrate the nature of nanoscale plasticity and to suppress the probability of microcracking at these biomedical ceramic parts. Further dedicated work is needed to determine the matter of which dynamic contact deformation takes place first in real biomedical contexts, given their complex geometry.

## 4. Conclusions

The nanoscale mechanical properties in ZTA ceramics fabricated by stereolithography-based additive manufacturing from ceramic paste were investigated by nanoindentation. On the basis of the shear stress active under the imprints, the critical load against the initiation of shear deformation band and/or microcracks in localized regions was clarified. The response of the phase boundary during indentation was emphasized through the crack propagation path, and optimization of alumina–YSZ adaptation through component design and SL processing was expected.

## Figures and Tables

**Table 1:** Comparison of the variation ranges for ZTA ceramics manufactured by AM and conventional technology.

Technology	Material	h_c_ (nm)	P_c_ (mN)	τ_max_ (GPa)	Ref.
AM	ZTA10	5.06–14.20	0.06–1.55	8.64–24.66	This work
Uniaxial pressing	10 ZTA (10 vol. %)	0.93–3.24	0.11–0.16	14.59–16.66	[30]
AM	ZTA30	2.85–13.42	0.04–1.82	7.42–24.81	This work
